# PERFICT: A Re‐imagined foundation for predictive ecology

**DOI:** 10.1111/ele.13994

**Published:** 2022-03-22

**Authors:** Eliot J. B. McIntire, Alex M. Chubaty, Steven G. Cumming, Dave Andison, Ceres Barros, Céline Boisvenue, Samuel Haché, Yong Luo, Tatiane Micheletti, Frances E. C. Stewart

**Affiliations:** ^1^ 8431 Pacific Forestry Centre Canadian Forest Service Natural Resources Canada Victoria British Columbia Canada; ^2^ 8166 Faculty of Forestry Forest Resources Management The University of British Columbia Vancouver British Columbia Canada; ^3^ Département des sciences du bois et de la forêt Pavillon Abitibi‐Price, 2405, rue de la Terrasse Université Laval Québec City Québec Canada; ^4^ 6314 FOR‐CAST Research & Analytics Calgary Alberta Canada; ^5^ 98686 Bandaloop Landscape‐Ecosystem Services Ltd. Nelson British Columbia Canada; ^6^ Canadian Wildlife Service Environment and Climate Change Canada Yellowknife Northwest Territories Canada; ^7^ Forest Analysis and Inventory Branch BC Ministry of Forests Victoria British Columbia Canada; ^8^ University of Victoria School of Environmental Studies Victoria British Columbia Canada; ^9^ Department of Biology Wilfrid Laurier University Waterloo Ontario Canada

**Keywords:** computational workflows, cross‐disciplinary, ecological forecasting, FAIR data, open models, predictive ecology, predictive validation, science‐policy integration

## Abstract

Making predictions from ecological models—and comparing them to data—offers a coherent approach to evaluate model quality, regardless of model complexity or modelling paradigm. To date, our ability to use predictions for developing, validating, updating, integrating and applying models across scientific disciplines while influencing management decisions, policies, and the public has been hampered by disparate perspectives on prediction and inadequately integrated approaches. We present an updated foundation for Predictive Ecology based on seven principles applied to ecological modelling: make frequent Predictions, Evaluate models, make models Reusable, Freely accessible and Interoperable, built within Continuous workflows that are routinely Tested (PERFICT). We outline some benefits of working with these principles: accelerating science; linking with data science; and improving science‐policy integration.

## Graphical abstract

To date, our ability to use predictions for developing, validating, updating, integrating and applying models across scientific disciplines while influencing management decisions, policies, and the public has been hampered by disparate perspectives on prediction and inadequately integrated approaches. We present an updated foundation for Predictive Ecology based on seven principles applied to ecological modelling: make frequent Predictions, Evaluate models, make model workflows Reusable, Freely accessible and Interoperable, built within Continuous workflows that are routinely Tested (PERFICT). We outline some benefits of working with these principles: accelerating science; linking with data science; and improving science‐policy integration.

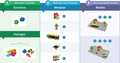

**Problem Statement**

Applied ecology faces time‐sensitive problems such as species declines, changes in primary productivity, and biological invasions. Processes advancing ecological understanding and weaving current science into management decisions and policies addressing these problems, often proceed too slowly and are more subjective than they could be. This is, in part, due to models that are weakly linked to data, challenging to reconfigure and improve, not readily connected to other models and disciplines, not designed for iterative forecasting, limited use of data science advances, and infrequently evaluated. We present a framework for Predictive Ecology that facilitates speeding up of inferential advances and model usefulness because of more rapid transferability.

## INTRODUCTION

The current biodiversity crisis and increasing pressures on socio‐ecological systems (e.g., climate, land use, and pollutants) present time‐sensitive challenges to ecosystem and landscape management, and sustainable development (McPhearson et al., 2021). As a result, the number of applied ecological models has exploded in recent decades (e.g., repositories for NetLogo Wilensky, 1999; EwE Christensen & Walters, 2004) and calls for iterative forecasting have been widely embraced (Lewis, Woelmer, et al., 2021). A wider application of these models would likely help to solve these challenges, as this would facilitate comparing models, building model ensembles, and testing hypotheses (Belete et al., 2017; Wenger & Olden, 2012). Yet the process of transferring models to new contexts (Yates et al., 2018) or across disciplines involves transferring *workflows* (Fer et al., 2021)—not just a model's mathematical components. These workflows potentially comprise many algorithmically rich steps including data assimilation, model parameterisation, fitting, prediction and assessment, and one or many output treatments. Transferring these workflows remains onerous because they tend to be either incomplete, inflexible or obscure.

Predictive Ecology, a branch of ecology based on quantitative deductions from models (Houlahan et al., 2015; McGill et al., 2007; Mouquet et al., 2015; Peters, 1977, 1991; Travers et al., 2019), provides a framing that helps with these challenges. However, it falls short in its current form; it is focused on models *per se*, not the workflow involved in generating predictions (Fer et al., 2021; Lewis, Woelmer, et al., 2021). Prediction is important in model evaluation (e.g., cross‐validation) and is important in forecasting (i.e., predictions of future conditions). Predictions from multiple models allow us to quantify impacts of different model assumptions and algorithms (e.g., Fajardo et al., 2020). Importantly, evaluating model fit by comparing model predictions to out‐of‐sample data allows comparisons of models from any paradigm—simulation, Bayesian, Machine Learning, likelihood, mathematical, etc.—because such comparisons are based on a chosen dataset *to compare with*, not the paradigm or data used for the original model. Yet, comparing models across studies for applied decision‐making occurs infrequently (though see Lewis, Rose, et al., 2021). We need the best models of appropriate complexity (Aho et al., 2014; Anderson et al., 2000; Horne & Garton, 2006; Wood et al., 2020) for each application (Dietze, 2017), even for cases where we do not have the resources of unified global efforts (e.g., IPCC: Masson‐Delmotte et al., 2021). Furthermore, the generality of models can be determined by how well they predict in many contexts. The science‐policy interface, on the other hand, needs ecologists and their models to be nimble enough to adapt for real‐time engagement needs of stakeholders (Ferraz et al., 2021). Ecology needs a framework that enables the transferability of each component of the modelling workflow and makes cross‐study evaluations rapid and commonplace. *We need to reduce the marginal effort of running models outside of their original study*.

Transferring models requires new datasets that match the structure of the originals, an understanding of the model, its implementation, the type of relationships in question, and attention to avoid inappropriate extrapolation beyond the original data (Yates et al., 2018). This becomes easier when each step of the original model workflow is modular, reusable, freely available, transparent and interoperable—i.e., the next application can reuse one or more components. For example, with multiple models of wildfire forecasts, each one may have a published study‐specific assessment of model fit. Yet, prediction quality for a new challenge is unknown: one might be more accurate at forecasting near human habitations, while another at forecasting peatland fires that accelerate permafrost melt.

**FIGURE 1:**
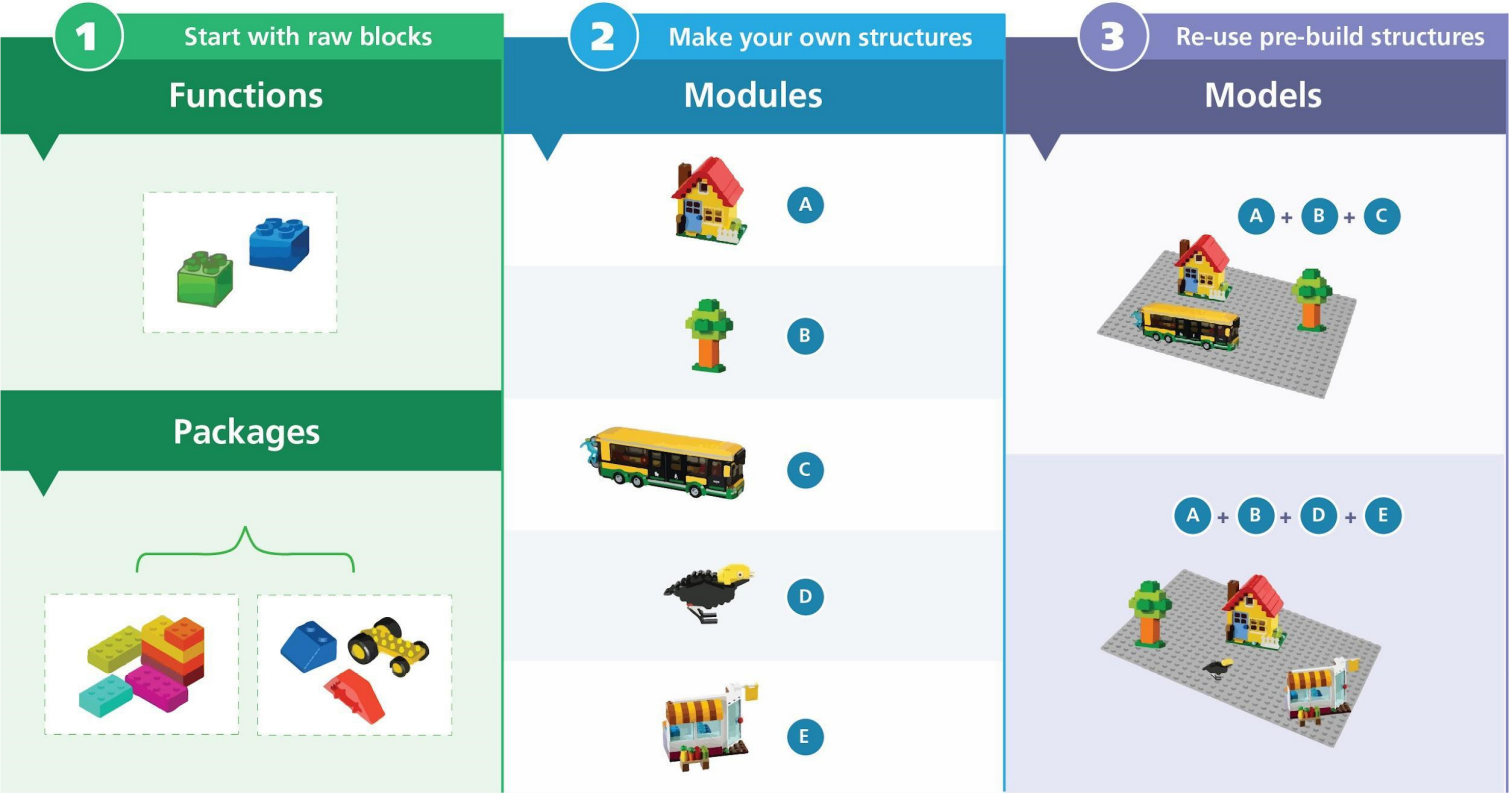
Functions and modules as key tools of a PERFICT approach. Functions are modular and can be bundled into packages that can utilise tools that enable easy dissemination, quality control, continuous integration, documentation, and writing. Functions may have default values for arguments, but they are not intended to do something without the user understanding the function and providing input arguments. Like functions, modules have inputs and convert those inputs into some output. However, modules are higher‐order collections of one or more functions that have computer and human readable metadata describing their inputs and outputs. Unlike functions, module metadata contain the information that describes how modules fit (or not) together. Modules, as we suggest here, are the basic unit of code that enables and facilitates all the elements of the PERFICT approach. In analogy, functions are Lego® pieces, often supplied in a package (collections of functions) with instructions (function documentation), and modules are Lego® structures made with those pieces (i.e., the original developer wrote the documentation and built the structure), such as trucks, houses, roads, space shuttles. A given structure has inherent value, e.g., a truck can be the end goal of a project and can be stand alone. The metadata (implicit in Lego®) describe the ways these structures interact, e.g., a road can take things with wheels (input); a bus has wheels (output), so can go on a road, but a house does not so cannot. Using a structure by itself or combining multiple structures together makes simple to complex “models”, such as neighbourhoods, villages, cities, or space stations. Many modules fit together (a truck and a road); others do not (a truck and a space station). The structures can be used in many new ways, bricks added to structures, and collected into complex meta‐structures. If we want to build a Lego® city, we could either start with individual bricks to build a new configuration or reuse some or all pre‐existing structures. Furthermore, other toy “brands”—or computer languages, e.g., R, Python, C++—can be added to the city. Using the PERFICT approach, ecologists build robust, reusable modules, enabling rapid creation, use, testing and reformulating of models

We propose a new foundation for Predictive Ecology that focuses on improving transferability through modularising the steps of ecological modelling workflows (Figure 1; Supporting Information B). This will enable better decision‐making based on science (Table 1; Supporting Information C). Here, we focus on presenting the concepts, yet toolkits (Chubaty & McIntire, 2021) exist that enable implementing these ideas (e.g., Micheletti et al., 2021; Supporting Information D).

**TABLE 1:** Benefits and examples of the PERFICT approach and how these benefits can be realised

Benefit	Example	PERFICT approach enables the benefit by:
Accelerating science	Occam's razor	Evaluation of how much complexity is right for a given project, as models of arbitrary complexity can be readily compared
	Informative priors	Easing the process of moving from a previous study's Bayesian posteriors to a new study's priors, lessening the problems with specifying uninformative priors (Northrup & Gerber, 2018)
	Forecast horizon	Repeatedly iterating a forecasting model with regularly updated data and model (Petchey et al., 2015)
	Community of contributors	Allowing manageable projects with hundreds of contributors to quickly update our understanding of a system (Fer et al., 2021)
	Predictive validation	Using future out‐of‐sample data to test models becomes easier with reusable, interoperable modules (Power, 1993)
	Rewriting models	Encouraging reimplementation in a widely known language (e.g., R) allowing many experts to see and understand code (Thiele & Grimm, 2015)
	Many eyes	Modelling standards that are understandable by many scientists with sufficient capacity to more readily fix bugs and identify improvements
Bridging to Data Science	Building on data science tools	Facilitating the use of cloud computing and repositories, user access control and data caching, for researchers who do not have the capacity or time to learn and develop them
	Data quality and quantity	Building data‐model‐validation pipelines from reusable components allowing for assessment of different data sources (White et al., 2019)
	Linking models to data	Maintaining linkages between canonical data sources and models live at all times allows for rapid reparameterisation and updating with continuous testing (Micheletti et al., 2021)
Improving science‐policy integration	Cross disciplinarity	Lessening the technological, data and cultural barriers that make cross‐disciplinary work challenging (Chassé et al., 2020)
	Regular reporting	Reducing the effort required to produce regular updates for policy reporting
	IPCC‐like process	Allowing lower budget projects to achieve IPCC‐like integration with its benefits such as regular updating, ensemble modelling, and contributions to policy (Masson‐Delmotte et al., 2021)
	Different users	Creating a complete framework that allows for all types of expertise—from land managers, rights holders and the public, to scientists and computer programmers—to interact (Ferraz et al., 2021)
	Web and decision support applications	Allowing for the development of generic web and decision support tools—“dashboards”—that can be reused widely
	Coping with contradictions	Opening the science informed decision‐making and policy‐making process to shed light on cases where models contradict one another and offering an objective way to resolve those contradictions

See Supporting Information C for further discussion. In each example, there may be certain elements of the PERFICT approach that may be more relevant; for clarity, we do not specify individually. In all cases, the more elements of the PERFICT approach that are followed by a model, the more beneficial the outcome.

## THE PERFICT APPROACH

The PERFICT approach for Predictive Ecology provides a foundation of seven principles applied to the ecological modelling workflow: make frequent Predictions, Evaluate models, make components Reusable, Freely accessible and Interoperable, built within Continuous workflows, that are routinely Tested. In doing this, we unify disparate components from computer science and forecasting and add elements that are unique to ecological modelling.

### Predict frequently

For ecologists to improve the quality of predictions, making models that have good assessments of statistical fit is insufficient; we must make and learn from *many* predictions by comparing to out‐of‐sample data (Lewis, Woelmer, et al., 2021; Tetlock & Gardner, 2016). Forecasting challenges are forcing some ecologists to do this (e.g., the Ecological Forecasting Challenge with NEON; https://ecoforecast.org/efi‐rcn‐forecast‐challenges/). Ecologists will also benefit from workflows that can be transferred to other contexts because they may gain access to new data (Barros et al. in review). The diminishing returns that may come from iterative improvements for any specific model should, however, be compared against costs of, e.g., particularly large models (Bender et al., 2021).

### Evaluate

The quality of a model's predictions (Milner‐Gulland & Shea, 2017) is not absolute. More accurately, a model can be sufficient for a current need (Rykiel, 1996). Indeed, estimating model fit with different data is one of several explanations for results not being reproducible (Baker, 2016). Thus, evaluating model predictions, especially with out‐of‐sample data, can be more effective at understanding quality, overfitting, and biases (especially egregious ones, Bender et al., 2021), and may improve ecological understanding (Power, 1993). When model workflows are interoperable, generic validation modules can be developed to compare multiple models more quickly (e.g., Barros et al. in review), and transferring models to new contexts can help with situations with insufficient data for validation. Validation approaches developed by numerous forecasting efforts (Lewis, Woelmer, et al., 2021) could be more broadly applied using reusable and modular workflow steps.

### Reusable

Reusability is the ability to, without the assistance of original developers, use modular components that comprise the modelling workflow, from the first steps of data importing through to output treatments. Component reusability means that if a study develops new methods (e.g., for model validation of a commonly used simulation model or for converting large global datasets to inputs for a model), other applications can reuse them, reducing effort required (Wenger & Olden, 2012). Reusability comprises five characteristics: each step must 1) be scripted; 2) produce the same answer with the same inputs (including random number generator seed, if stochastic); 3) produce a different, but equivalent, answer with different inputs; 4) work on all common computer platforms; and 5) have meta‐information (metadata) describing how it can interact with other components. *Reproducibility* (Borregaard & Hart, 2016)—a special case of *reusability*—can be achieved with characteristics 1 and 2 (Baker, 2016; Begley & Ellis, 2012; Klein et al., 2014; Munafò et al., 2017). The first four can be efficiently developed by creating functions, wrapped in packages and hosted in open repositories (e.g., https://cran.r‐project.org/; https://pypi.org/). However, a collection of functions is insufficient to solve a particular task because the required sequence of steps does not emerge from the functions (Figure 1). Additional metadata is required, defining how functions interact with the environment that calls them. In practice, ecologists can bundle sequences of functions into meaningful modules (e.g., “data preparation”, “parameter estimation”, “climate sensitive fire simulation”; Figure 1 and Supporting Information Fig B1; see Micheletti et al., 2021) which will range from specific to generic, and combine them with those of other creators. The sequencing of these modules can emerge from this metadata (Supporting Information Fig D1), similar to how software package managers determine the installation order of packages and their dependencies.

### Freely accessible

Open science and free, available, interoperable and reusable data accelerate innovation, as well as improve transparency and accountability (Reichman et al., 2011; FAIR: Stall et al., 2019; ART: Bodner et al., 2020 Supporting Information A). Developing open modelling workflows (including collaborative version control systems, such as https://github.com) also allows other scientists to evaluate the implementation of the science. While performance tradeoffs exist, using programming languages that are widely used by ecologists (e.g., currently R, Python, Julia) can make models even more accessible, transparent, readily (re‐)usable and testable by others (Lai et al., 2019; see Accelerating Science Supporting Information C).

### Interoperable

Interoperability embodies modularity *and* standards. Modularity arises when a description of a component has structured, human‐ and machine‐readable metadata (Figure 1). The two most important design criteria are that modules should 1) be able to run either independently or as a subcomponent of a larger model, and 2) communicate with other modules via their inputs and outputs (Reynolds & Acock, 1997; Voinov et al., 2004). To ensure modular pieces are interoperable, they must follow standards that define how modules communicate. Modules with metadata for inputs and outputs, and developed in widely used programming languages increase interoperability of model components (Belete et al., 2017).

### Continuous workflow

Recently, authors have advocated for continuous workflows for near‐term forecasting (Dietze et al., 2018; White et al., 2019). These workflows are just as useful in other contexts, such as policy development and strategic land management planning (Paradis et al., 2013), or predicting in new situations. To implement a continuous workflow, ecologists generally build scripts with e.g., data loading, compiling, estimation, validating and reporting. When the individual steps of the workflow are reusable, the workflow can become both modular and continuous, facilitating rapid iterations for a given study, and rapid sharing of components across studies (Fig B1). Since long computational steps are common in ecology and researchers want to run these only once, functions that are deemed too intensive to rerun frequently require caching (e.g., McIntire & Chubaty, 2021; Micheletti et al., 2021) to maintain continuous workflows even for very complex models and ensure breakages are identified quickly.

### Testing automatically

We distinguish two parts of testing: ecological validation (“Evaluation” described above) and code testing. The objectives of code testing include evaluating code efficiency, detecting errors in algorithm implementation, and translating mathematics to code. Robust approaches come from software development fields and include using code assertions (Rosenblum 1995) and writing unit, integration and system tests (Scheller et al., 2010). Attaching ecological validation and/or code testing to automated continuous integration (CI) systems is straightforward, e.g., GitHub Actions (https://docs.github.com/en/actions) for small projects (e.g., Barros et al. in review) or individual components, and advanced research compute platforms for larger problems.

## CONCLUSION

The future of modelling in applied ecology requires transferable solutions of all the components of workflows that cross disciplines and transcend scientific, statistical, computational, and cultural paradigms (e.g., Micheletti et al., 2021). Some solutions for applied problems have reflected elements of the PERFICT approach (Geller & Turner, 2007; Parrott, 2017), but these successes are too rare (Travers et al., 2019). The PERFICT formalisation modular workflows, facilitating cross‐study model comparison, hypothesis testing, and ensemble modelling, while promoting utility, flexibility, adaptability and scientific longevity because they can be easily rerun by the ecological community (Reynolds & Acock, 1997; Table 1; Supporting Information C). This creates robust and nimble models for a range of ecological applications including iterative forecasting cycles (Dietze et al., 2018). Ecologists are embracing modern predictive approaches (Lewis, Woelmer, et al., 2021), benefitting decision‐ and policy‐making for ecosystems worldwide. The PERFICT formalisation can facilitate data‐model integration, and tighten science‐policy integration, because the nimbleness that can come from reusable and interoperable modules for a modelling workflow allows science to respond rapidly to changing policy demands (Table 1; Supporting Information C and D). Reducing the friction of transferring other model workflow components will make it easier to evaluate and improve models, taking us more quickly to the best models for today's challenges.

## AUTHORSHIP

EM, AC, SC conceived of the idea; EM wrote the first draft; DA and SH iterated through the PERFICT approach for management applications; all authors contributed substantially to the ideas; EM, AC, CBa, CBo, YL, TM, FS contributed substantially to revisions.

### PEER REVIEW

The peer review history for this article is available at https://publons.com/publon/10.1111/ele.13994.

## Supporting information

Supplementary Material

## Data Availability

No data were used in this study. All models and code mentioned are freely available and hosted on GitHub or on the Comprehensive R Archive Network, with their links provided in the text.
