# Supplementary material for: PERFICT: A Re‐imagined foundation for predictive ecology
Source: Ecol Lett. 2022 Mar 22;25(6):1345–51. doi: 10.1111/ele.13994 (PMC9310704; doi:10.1111/ele.13994)
Supplement: Supplementary file 1 — Supplementary Material [file ELE-25-1345-s001.docx]

# Supplemental Material

### A Mapping of PERFICT, FAIR and ART

Several acronyms have been developed to help researchers create more productive, open science. These each address slightly different aspects of ecological data and modeling, though they do have some overlapping applications.

| PERFICT (models) | FAIR (data: Stall *et al.* 2019) | ART (models: Bodner *et al.* 2020) |
| --- | --- | --- |
| **P**redict frequently | -- | -- |
| **E**valuate | -- | **A**ccurate, **R**eliable |
| **R**eusable | **R**eusable | **T**ransparent |
| **F**reely accessible | **F**ree & **A**vailable | **T**ransparent |
| **I**nteroperable | **I**nteroperable | -- |
| **C**ontinuous workflow | -- | -- |
| **T**ested Automatically | -- | **A**ccurate |

###

###

### B Modular Workflows


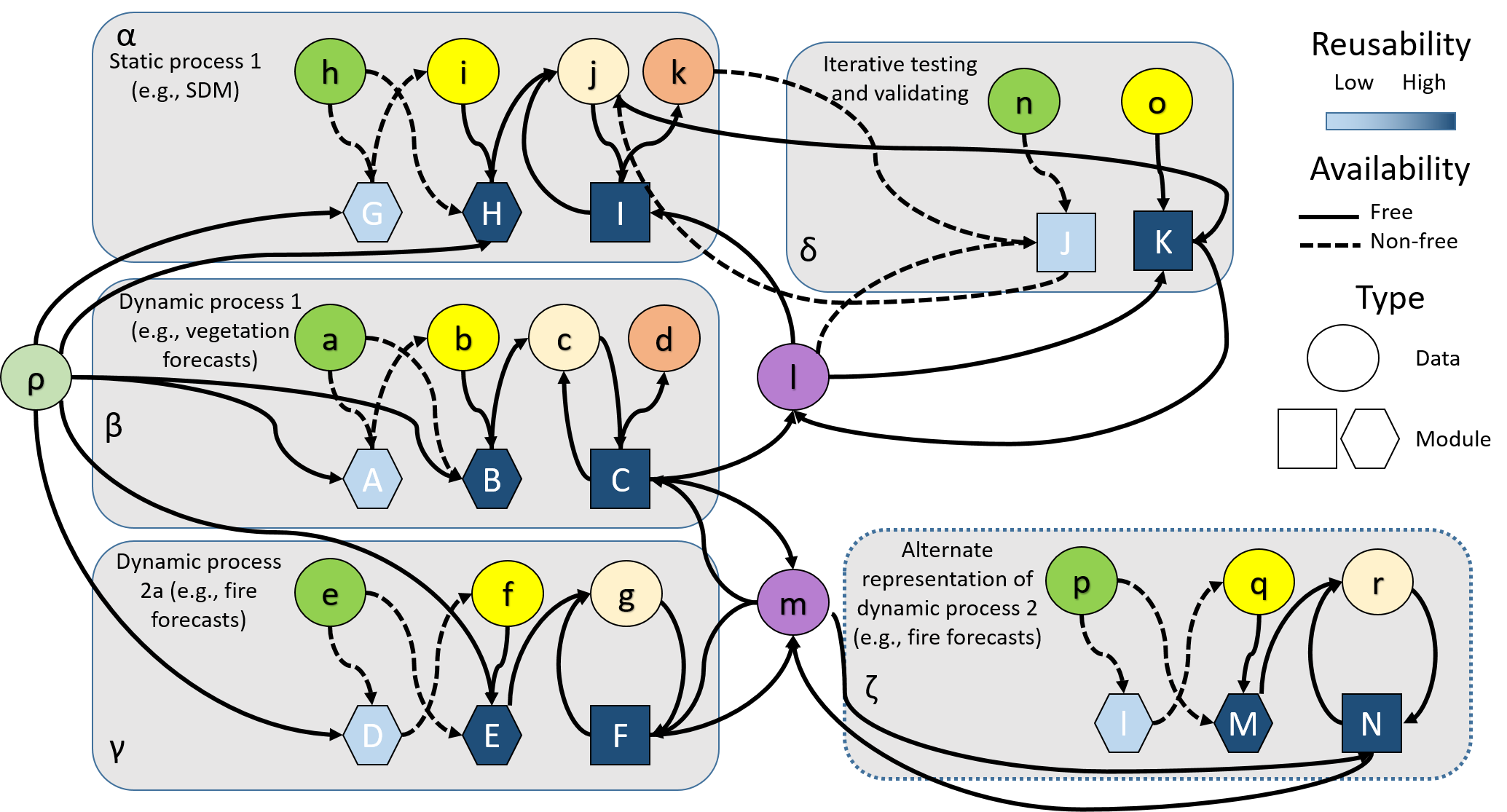


Fig. B1. Example abstraction of a PERFICT approach to a study. In this view, the study can be nimble as many components are reused, the entire workflow is continuous, changing data availability is accommodated, alternative modules for the same process are explicit, each sub-project has high modularity (few arrows cross sub-project boundaries), and there are very few data sources that are external to each module demonstrating that the cross-disciplinary connections are minimal. For a given sub-project, a ubiquitous workflow is to have 2 generic modules (one for parameter estimation -- hexagon -- and one for forecasting or predicting -- square), with zero or more idiosyncratic modules. In cases of maximum reusability, ρ is the only idiosyncratic dataset (i.e., is specific to this project, with no anticipated re-use) that must be supplied. In our experience, each project begins with many idiosyncratic datasets and non-reusable modules to deal with those idiosyncratic datasets, in part because we do not yet know what is reusable. But as we identify the components that are reusable, over time and use, more and more elements move from idiosyncratic modules to generic modules (e.g., elements in module G are moved to H). Similarly, as all the data in a project become freely accessible, the idiosyncratic modules may be dropped, simplifying the project, and maximizing reusability. We include an alternative collection ζ that represents the same ecological process as γ; the two together can inform “consensus” forecasts, be treated as alternative hypotheses, help to estimate model uncertainty etc. Modules J and K can be built to provide feedbacks into any arbitrary modules. Where there is a need to use heuristic optimization (e.g., pattern oriented modeling: Grimm & Railsback 2012), a single objective function can be developed to update arbitrary parameters (not shown). A traditional ecological study would include h and G, i.e., closed data and low reusability models. Arrows represent data-module connections with freely (solid lines), and not freely (dashed) available data. Greek letters indicate sub-projects which are collections of modules that create a coherent data-module workflow for a single idea (e.g., “wildfire forecasting”), which could be from a scientific publication. Alphabet letters are arbitrary labels for data (lowercase) or modules (uppercase) showing that data input expectations will generally (though not necessarily) be unique for a given module. Within the data types, green is proprietary or truly idiosyncratic data, yellow is freely accessible and open data, beige is data inputs and (possibly) outputs of modules, orange is outputs that are not inputs (e.g., for visualization, reporting etc.), and purple is shared data. We show data in different colors to emphasize their different roles; within a project, they are simply arbitrary data objects. Within the module types, the darkness of the coloration indicates how generic it is, therefore how reusable it is in different contexts. ρ is the study area for a project.

### C Benefits of PERFICT approach

*Accelerating science*. One of the primary objectives for model estimation as a process that advances science is to avoid overfitting (e.g., via approaches such as AIC). However, minimizing overfitting by using a single dataset (e.g., cross-validation, AIC) will have limited success (Reunanen 2003). The more independent the data are, the greater success in minimizing overfitting (e.g., predictive validation Power 1993; Reunanen 2003; Wenger & Olden 2012). Nevertheless, the widespread use of independent data for fitting/validating is limited, because, we believe, most models and projects are not ready when independent data become available, particularly if the models have complex data requirements. Using modular, reusable and interoperable models can greatly contribute to speeding up scientific advances by enabling faster and iterative re-evaluation and updating of these models (and model fit) -- by the original model creators or others -- when new data become available for validation and/or prediction. As the number of such models grows, models can routinely become part of meta-model comparisons and the appropriate level of complexity can be determined. This will help overcome the “dinosaur problem of simulation models,” where models get “bigger, bigger, bigger, useless” (H. Kimmins, pers. comm.) because there is always another process that seems critical to include. Over time, forecast success from models will improve and the forecast horizon will extend outwards (Petchey et al. 2015). Furthermore, scientists will have access to complete model objects (e.g., *sensu* R language), to which statistical and graphical “methods” (e.g., R functions like predict, AIC, drop) can be applied -- instead of tables of coefficients -- from published work. Using fully functioning models from other researchers, we gain more power and flexibility for forecasting, for iterative improvements (e.g., because they contain the variance-covariance structures), for meta-modelling and testing alternative hypotheses (e.g. ensemble or consensus forecasts; Marmion et al. 2009), and even near-automatic meta-analyses across studies and systems (Hedges *et al.* 1999; Koricheva *et al.* 2013). Finally, rewriting widely used models, while labour intensive, can be profitable for the broader community through increased interoperability and reusability (Thiele & Grimm 2015). A community of contributors accelerates the implementation of new insights (e.g., data inadequacies, ecological processes) and helps discover and fix bugs (Barros *et al.* in review) and with internal modularity building and adding new components will be easier in the future. With the PERFICT approach, every project can be its own collection of modules; modules can be added, removed, adjusted depending on their (automatic) testing against data. This modular complexity is the practical implementation of Occam’s razor.

*Bridging to Data Sciences.* One of the reasons to adopt the PERFICT approach is to build formalization and thus gain powerful tools from neighbouring data science field – i.e. pipelines (Beaulieu-Jones and Greene 2017), online databases and repositories (using or building), online services (e.g., Google Earth Engine, Moore and Hansen 2011), online data visualization (e.g., leaflet; Crickard III 2014) and web applications (e.g., shiny; Chang et al. 2019) – which can be algorithmically linked throughout a project. These links can be made by a data-savvy scientist, built into functions, packages and modules, and then used more broadly. For example, user access control (UAC) is a reality for many datasets: not all datasets are yet FAIR (Stall *et al.* 2019). Developers can use UAC tools (e.g., Google Authentication) without breaking the continuous workflow. When a new user downloads a module, the module automatically downloads the data it needs, assisting the user with advanced tools such as checkpointing and spatial cropping, projecting, masking and data integrity checking (see *prepInputs* function in the *reproducible* package). By maintaining the connection to the original data sources, a user can get updates as needed. These links between data and models also enable a quicker re-parameterization and re-validation against new data or when using the model in a new study area. Following the PERFICT approach, parameter estimation modules and validation modules can be developed and included as a part of a project to link both calibration data and validation data (See Supp. Mat. Fig. B1; Barros *et al.* in review), and continuous parameterization and validation can be realized. Furthermore, the PERFICT approach, e.g., via predictive validation, creates a formal and rapid way to let the data tell us which data are better for a particular question. This is particularly important in Ecology, where various data sources whose quality and quantity range widely, e.g., high quality but modestly sized field data vs. very large remote sensing datasets (of varying quality).

*Improving science-policy integration*. The approach used by the Intergovernmental Panel on Climate Change (IPCC) provides a template for science-policy integration. The IPCC brings together more scientists than a typical research project, runs many different models, and integrates model outputs to test hypotheses, to understand model uncertainty and divergent or common outputs, to build iterative forecasts of the future, and to compare data as the future represents a forecasting-based hypothesis test (<https://www.ipcc.ch/assessment-report/ar6/>). Replicating this approach for every applied ecological problem will require major improvements in how ecologists integrate across scientific disciplines and models, utilize large and novel data, and repeat this process. The PERFICT approach outlines a way to replicate the process of the IPCC (wholly or partially), but with vastly fewer resources. It encourages a nimble approach to applied decision making (Box 2) that allows for both changeable process complexity (e.g., a simple or complex fire model) and management complexity (e.g., manage wildfire risk in isolation, or within the context of forest management, species-at-risk, climate change and pest management). Scientifically, the easier testing of alternative models and hypotheses using the PERFICT approach offers an objective ground to resolve contradictions from models. From a management perspective, competing land management goals such as carbon sequestration and species at risk conservation can be evaluated, crossing traditional scientific disciplines and synergies can be identified. With new and more data, the predictions from potential models are checked against data, reported clearly and rapidly, and repeated regularly. This translates directly to policy spheres that have a regular reporting requirement (e.g., Stinson *et al.* 2011). It also brings decision making into a continuous improvement process, allows for the creation of generic decision support systems that can be customized for specific applications and builds confidence in science-informed decision-making. Finally, PERFICT improves science-policy integration by increasing model interoperability horizontally (e.g. integrating across disciplines) and vertically (Fig. C1). While the literature has a track record of this sort of science-policy integration happening (e.g., Schmolke *et al.* 2010), the PERFICT approach will allow this to become ubiquitous and speed the transfer of vertical information. This expands the reach of ecological models beyond ecologists and promotes co-production by enabling the direct participation and feedback of non-experts, like policy and decision makers.

###
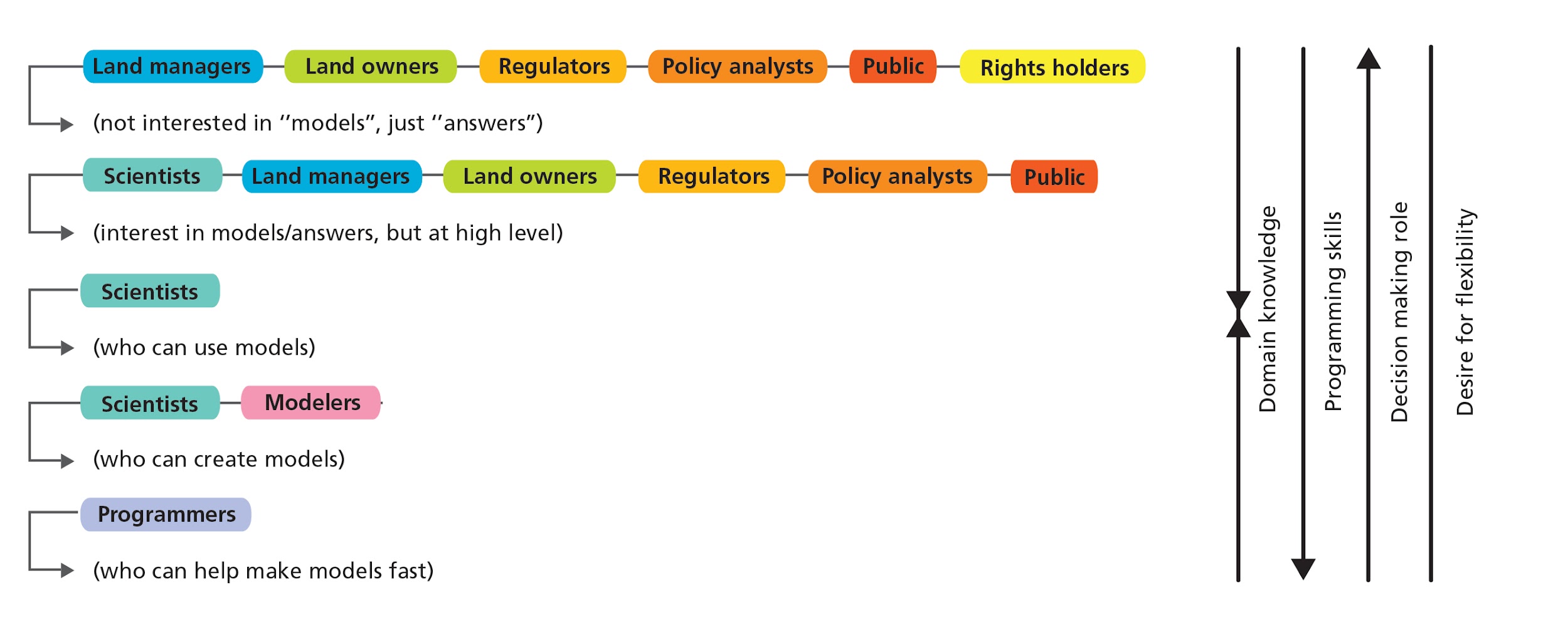


Fig. C1. The different users, and their contributions to applied decision making (arrow-heads show increasing importance), who interact with ecological modeling and their forecasts. The PERFICT approach modularity allows many entry points into a science-policy system. We used the SpaDES toolkit (Chubaty & McIntire 2021) in our projects, which enhances existing open data and tools, facilitates the implementation of this approach, and improves the ability to engage a wide variety of users.

###

### D PERFICT in action

Pushing against parsimony, it is now clear that we often need heterogeneous models that integrate across disciplines so that realistic land management challenges can be addressed (Houlahan *et al.* 2015). For example, land managers in Northwest Territories (NWT), Canada, are attempting to manage declining woodland caribou populations and listed bird species-at-risk in the context of protected areas planning and indigenous peoples’ rights (Micheletti *et al.* 2021). This formed the basis for a pilot project for the PERFICT approach. To forecast these values, there were many ecological and land management issues that had to be addressed. For example, wildfires had to be forecasted under changing climate, changing fuels (vegetation), and changing fire suppression practices. Vegetation is shifting due to direct and indirect effects of climate, such as species and biome shifts, permafrost melt, tree species drought-induced mortality, and accelerated forest succession dynamics, to name a few. These landscapes are also currently and historically inhabited and used by Indigenous peoples (<https://native-land.ca/maps/territories/sahtu-dene-and-metis/>). There are important road networks for mining and other anthropogenic development. There are enormous carbon stores in the frozen peatlands that are melting, releasing these to the atmosphere. The overarching question was how to best manage the Species-at-Risk, alongside all these other values, given changing climate and indigenous peoples rights.

To effectively manage these landscapes, all these elements must be included in forecasting, so decisions can evaluate consequences, synergies and trade-offs across multiple disciplines. It is likely inappropriate to give any of these issues short shrift and have each project treat the issues that are not well studied by the team as “externalities”; yet, building large collaborative projects with models that do not interoperate is extremely onerous, time consuming, and ultimately very costly. We need to focus on management problems, while including *all the best disciplinary models*. This challenge led to the creation of a new class of ecological scientist: the *integrator*. Like a traditional generalist, this person acts within the big picture perspective, yet has just enough knowledge of the modules (and potentially the community of scientists who developed them) to be able to work at the interface between modules.

For the pilot, we assembled a 24-member collaborative team and brought together 19 modules with 7 lead module developers to assist with this problem (see Supp. Mat. Fig D1). The team included partners from two levels of government, and scientists from three universities, and the initial pilot was pulled together in four months. With the PERFICT approach, the technical parts of linking the models were a minor component of the whole project. The challenges we faced were not from integration, as we were using interoperable modules (Chubaty & McIntire 2021), but from the immature science that some of the 19 modules addressed.

In the expansion of the pilot -- the “Western Boreal Initiative” -- we are working with over 40 modules (some reused from the NWT project), with 10 different sub-projects including endangered species conversation, Indigenous land management, the Pan-Canadian Approach to conservation (Environment and Climate Change Canada 2018), caribou management and carbon management. Some of these projects are addressing whole-system management questions, others are very specific. Model components were either new or were existing; many required improved algorithms or had access to improved datasets as compared to the pilot. In all cases, each benefited from working within the PERFICT approach, allowing for nimble updates at any point, swapping out of previous models, and weaving in new elements including the long term process of Indigenous rights on the land. These are co-produced, works in progress.


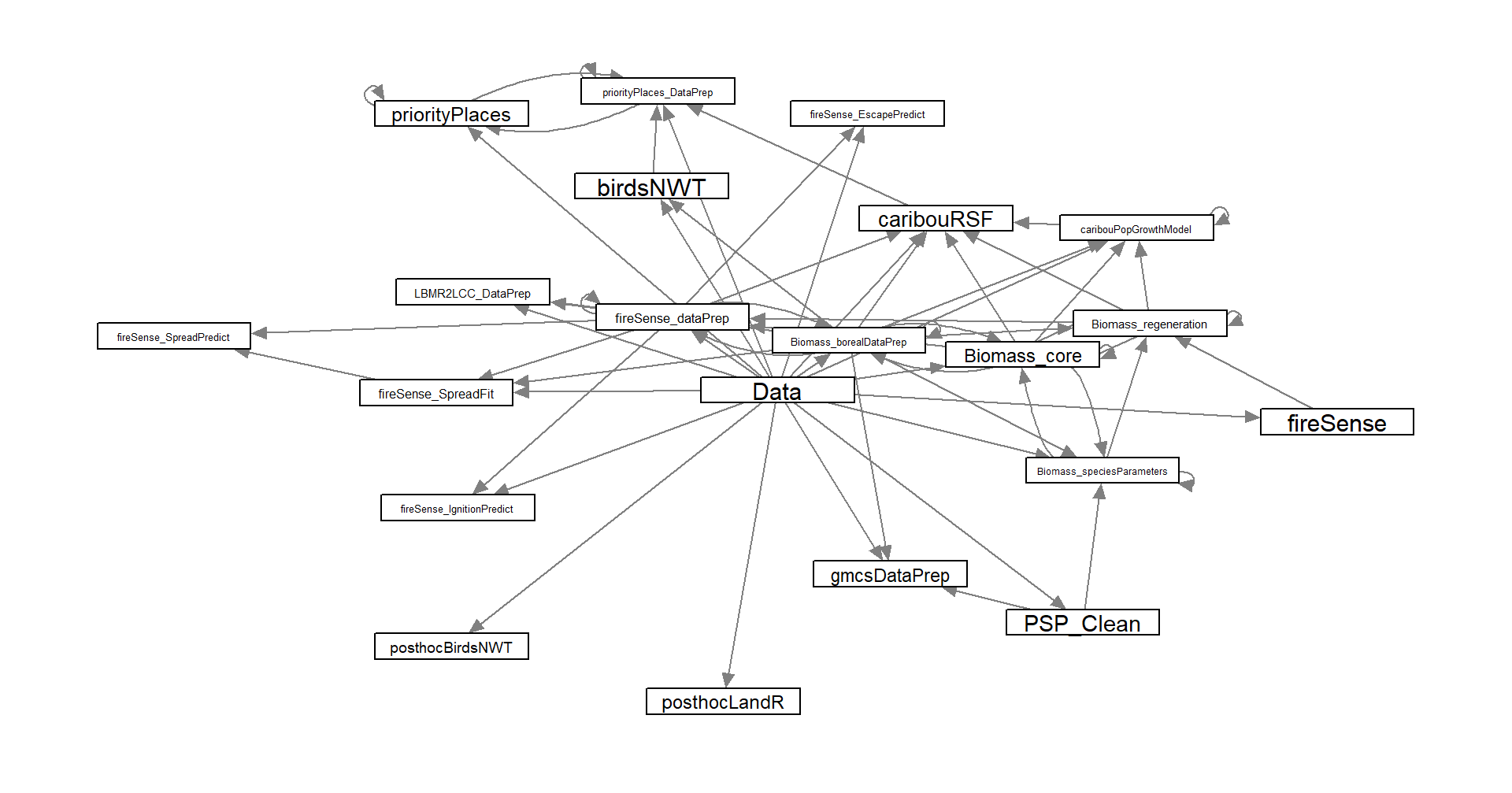


Fig. D1. An example of module interdependencies whose structure emerges from the metadata. These were used in Northwest Territories, Canada (Micheletti *et al.* 2021; Stewart *et al.* in review), demonstrating the linkage between shared inputs and outputs.

## References

Barros, C., Luo, Y., McIntire, E.J.B., Chubaty, A.M., Eddy, I., Andison, D.W., *et al.* (in review). Empowering ecologists in a simulation context: using R from data to complex landscape modelling, the LandR study case.

Bodner, K., Fortin, M.-J. & Molnár, P.K. (2020). Making predictive modelling ART: accurate, reliable, and transparent. *Ecosphere*, 11, e03160.

Chubaty, A.M. & McIntire, E.J.B. (2021). *SpaDES: Develop and Run Spatially Explicit Discrete Event Simulation Models. An R meta package*.

Environment and Climate Change Canada. (2018). *Pan-canadian approach to transforming species at risk conservation in Canada* ( No. ISBN: 978-0-660-27223-8). Gatineau, QC, Canada.

Grimm, V. & Railsback, S.F. (2012). Pattern-oriented modelling: a “multi-scope” for predictive systems ecology. *Philos. Trans. R. Soc. Lond. B. Biol. Sci.*, 367, 298–310.

Hedges, L.V., Gurevitch, J. & Curtis, P.S. (1999). The meta-analysis of response ratios in experimental ecology. *Ecology*, 80, 1150–1156.

Houlahan, J.E., McKinney, S.T. & Rochette, R. (2015). On theory in ecology: Another perspective. *BioScience*, 65, 341–342.

Koricheva, J., Gurevitch, J. & Mengersen, K. (2013). *Handbook of meta-analysis in ecology and evolution*. Princeton University Press.

Micheletti, T., Stewart, F.E.C., Cumming, S., Haché, S., Stralberg, D., Tremblay, J.A., *et al.* (2021). Assessing the pathways of climate change effects in SpaDES: an application with boreal landbirds in Northwestern Canada. *Front. Ecol. Evol.*

Power, M. (1993). The predictive validation of ecological and environmental models. *Ecol. Model.*, Theoretical Modelling Aspects, 68, 33–50.

Reunanen, J. (2003). Overfitting in Making Comparisons Between Variable Selection Methods. *J. Mach. Learn. Res.*, 3, 1371–1382.

Schmolke, A., Thorbek, P., DeAngelis, D.L. & Grimm, V. (2010). Ecological models supporting environmental decision making: A strategy for the future. *Trends Ecol. Evol.*, 25, 479–486.

Stall, S., Yarmey, L., Cutcher-Gershenfeld, J., Hanson, B., Lehnert, K., Nosek, B., *et al.* (2019). Make scientific data FAIR. *Nature*, 570, 27–29.

Stewart, F.E.C., Micheletti, T., McIntire, E.J.B., Haché, S., Leblond, M., Tremblay, J.A., *et al.* (in review). Forecasting caribou resource selection and demography under landscape and climate change in the Northwest Territories, Canada.

Stinson, G., Kurz, W.A., Smyth, C.E., Neilson, E.T., Dymond, C.C., Metsaranta, J.M., *et al.* (2011). An inventory-based analysis of Canada’s managed forest carbon dynamics, 1990 to 2008. *Glob. Change Biol.*, 17, 2227–2244.

Thiele, J.C. & Grimm, V. (2015). Replicating and breaking models: good for you and good for ecology. *Oikos*, 124, 691–696.

Wenger, S.J. & Olden, J.D. (2012). Assessing transferability of ecological models: an underappreciated aspect of statistical validation. *Methods Ecol. Evol.*, 3, 260–267.
